# Cx43 Isoform GJA1-20k Promotes Microtubule Dependent Mitochondrial Transport

**DOI:** 10.3389/fphys.2017.00905

**Published:** 2017-11-07

**Authors:** Ying Fu, Shan-Shan Zhang, Shaohua Xiao, Wassim A. Basheer, Rachel Baum, Irina Epifantseva, TingTing Hong, Robin M. Shaw

**Affiliations:** ^1^Cedars-Sinai Medical Center, Cedars-Sinai Heart Institute, Los Angeles, CA, United States; ^2^Department of Medicine, University of California, Los Angeles, Los Angeles, CA, United States

**Keywords:** gap junction protein, Cx43, alternative translation, mitochondria, microtubule, oxidative stress

## Abstract

Connexin 43 (Cx43, encoded by *GJA1*) is a cell-cell communication gap junction protein expressed in all organ systems. It was recently found that *GJA1* mRNA undergoes alternative translation to generate N-terminal truncated isoforms, of which GJA1-20k is the most abundant. Here we report a surprising finding that, unlike full length GJA1-43k, GJA1-20k has a strong tropism for mitochondria. Exploring function, we found that GJA1-20k appears to be an organelle chaperone and that overexpression of GJA1-20k is sufficient to rescue mitochondrial localization to the cell periphery upon exposure to hydrogen peroxide, which effectively limits the network fragmentation that occurs with oxidative stress. By high-resolution fluorescent imaging and electron microscopy, we determined that GJA1-20k is enriched at the interface between mitochondria and microtubules, appearing to load organelles for transport. Mutagenesis experiments revealed that although the microtubule-binding domain (MTBD) in GJA1-20k is not necessary for protein localization to mitochondria, the MTBD is essential for GJA1-20k to facilitate mitochondrial transport and maintain mitochondrial localization at the periphery. These results reveal an unexpected role for the alternatively translated isoform of the Cx43 gap junction protein, GJA1-20k, which is to facilitate microtubule-based mitochondrial transport and to maintain mitochondrial network integrity during cellular stress.

## Introduction

Connexin 43 (Cx43) is the most widely expressed of the 21 human connexins, occurring in all organ systems and is best recognized as a cell-cell communication protein (Beyer et al., 1987). Alternations of Cx43 expression and function are associated with numerous diseases including cardiac arrhythmias (Luke and Saffitz, 1991; Smith et al., 1991; Beardslee et al., 1998), cancer (Solan et al., 2012), ischemic injury (Beardslee et al., 2000), connective tissue disease (Paznekas et al., 2003), and heart failure (Akar et al., 2007; Basheer and Shaw, 2016). The mechanistic relationship between a cell-cell coupling protein and most non-arrhythmic diseases is not well understood.

Human Cx43 is encoded by the *GJA1* gene in which the entire coding sequence is within the second exon (Fishman et al., 1991). Thus, *GJA1* cannot produce different protein isoforms via alternative splicing. It was recently found that ribosomal translation initiating at internal AUG start codons within the mRNA coding sequence, commonly known as alternative translation, generates multiple endogenous carboxyl-terminal (C-terminal) segments of Cx43 protein. These products of alternative translation are N-terminal truncations of the full length Cx43 protein. Alternative translation of *GJA1* occurs widely in cell lines (Smyth and Shaw, 2013; Salat-Canela et al., 2014), primary cells (Salat-Canela et al., 2014; Ul-Hussain et al., 2014), and human heart (Smyth and Shaw, 2013). Mammalian target of rapamycin (mTOR) and MAP kinase-interacting serine/threonine-protein kinases 1/2 (Mnk1/2) signaling pathways (Smyth and Shaw, 2013; Salat-Canela et al., 2014) have been found to regulate alternative translation and inhibition of either mTOR or Mnk1/2 increases expression of the truncated isoforms (Smyth and Shaw, 2013; Salat-Canela et al., 2014). Pathway dependence implies alternative translation of *GJA1* can be turned on or off, depending on metabolic condition. Hypoxic stress is another condition that induces endogenous expression of the smaller isoforms (Ul-Hussain et al., 2014).

The role of the smaller alternatively translated isoforms of GJA1 is largely unknown. Of the isoforms, GJA1-20k is commonly the most highly expressed (Smyth and Shaw, 2013; Salat-Canela et al., 2014; Ul-Hussain et al., 2014). We have previously reported that GJA1-20k occurs in endoplasmic reticulum and aids full length Cx43 trafficking to cell-cell borders (Smyth and Shaw, 2013; Basheer et al., 2017), indicating that GJA1-20k can act as a chaperone in forward trafficking.

In this study, we report the surprising finding that in additional to full length Cx43 trafficking, GJA1-20k also targets to mitochondria. Furthermore, upon H_2_O_2_-induced stress, GJA1-20k but not full-length Cx43 is sufficient to preserve localization of mitochondria at the cell periphery. Using electron microscopy, we identified GJA1-20k at the mitochondria and the interface between microtubules and mitochondria, appearing to load mitochondria for transport. Mutagenesis experiments reveal that the previously identified tubulin-binding domain in GJA1-20k (Saidi Brikci-Nigassa et al., 2012) is not required for its mitochondrial localization but is essential for rescue of mitochondrial network integrity upon H_2_O_2_ treatment. In addition, under control conditions, GJA1-20k promotes microtubule-based mitochondrial transport, which is lost when the tubulin-interacting residues are removed. Taken together, these findings introduce the alternatively translated GJA1-20k isoform of the traditional Cx43 gap junction protein as a regulator of microtubule-based mitochondrial transport, which protects the organelle network during stress.

## Materials and methods

### Molecular biology

Human *GJA1* cDNAs (Open Biosystems) encoding full-length Cx43 and small isoforms were first cloned into pDONR/221 to generate Gateway entry clones (Thermo Fisher Scientific) as previously described. Destination vectors (pDEST) encoding C-terminal GFP-, mCherry-, and HA-tagged proteins were subsequently made for mammalian cell expression. All constructs are driven by the cytomegalovirus (CMV) promoter, and contain internal methionine start sites mutagenized to leucine to ensure single isoform expression. Mutagenesis was carried out with the Quick Change Lightning Mutagenesis Kit (Agilent) according to manufacturer's instructions. Mutagenesis primers used to remove all six tubulin-interacting residues (Saidi Brikci-Nigassa et al., 2012) in GJA1-20k-del6 are: 5′ AAG GGC GTT AAG GAT AAG GGA AAG AGC GAC CCT AGT GGT GCG CTG AGC 3′, and 5′ GCT CAG CGC ACC ACT AGG GTC GCT CTT TCC CTT ATC CTT AAC GCC CTT 3′. The mito-BFP plasmid was a gift from Dr. Gia Voeltz (Addgene plasmid # 49151).

### Cell lines and primary cell isolation

HeLa, HaCaT, and HEK293T cell lines (ATCC) were maintained at 37°C in a humidified atmosphere of 5% CO_2_ in fully supplemented media containing DMEM with 10% fetal bovine serum (FBS), nonessential amino acids, sodium pyruvate (Thermo Fisher Scientific), and Mycozap-CL (Lonza). Primary cardiac fibroblasts were isolated from neonatal mouse hearts as described (Zhang et al., 2014). Fibroblasts adhering to the dish during the pre-plating step were enriched for 1 week in DMEM:F12 supplemented with 3.5% FBS and 1x Mycozap-PR (Lonza). Primary glial cells were isolated from neonatal mouse brains as previously described (He et al., 2007; Habas et al., 2013).

### Biochemistry

Co-immunoprecipitation was carried out using HeLa cells expressing HA-tagged GJA1-20k. Cytoskeletal-protein interactions were stabilized by the addition of phalloidin (25 μM, Sigma-Aldrich) during cell lysis in 0.5% NP40 buffer (in mM, 150 KCl, 20 HEPES, 2 MgCl_2_, 2 K_2_HPO_4_, 1 DTT, 1 NaF, 0.1 Na_3_VO_4_, 0.5% NP40, pH7.4 with halt protease inhibitor). Cells were rotated for 30 min and spun down at 10,000 × g for 20 min at 4°C to remove insoluble debris. Following protein normalization, cell lysates were precleared using Dynabeads protein G (Thermo Fisher Scientific) for 30 min with rotation at 4°C. Beads were discarded, and 2 mg of precleared lysate was used for each reaction. Immunoprecipitation was undertaken using 5 μg of either mouse anti-HA (4C12, Abcam), or mouse anti-GST (B-14, Santa-Cruz Biotechnology) as negative isotype control at 4°C for 4 h with rotation. Dynabeads protein G (20 μl) was added to each reaction, and the tubes were rotated for an additional 45 min at room temperature. Protein complexes were washed 3 times with lysis buffer using a Dynamag-2 magnet. Proteins were then eluted with 30 μl 2X NuPAGE sample buffer containing 100 mM DTT, incubated at 37° for 20 min, and subjected to SDS-PAGE electrophoresis and Western blotting. Membranes were imaged using the ChemiDoc MP detection system (Bio-Rad). The experiments were repeated three times.

### Mitochondrial fractionation

Mitochondria fractionation of HeLa cells transfected with HA-tagged GJA1-43k or GJA1-20k, was performed as previously described (Singh et al., 2012). Cells were manually homogenized in isolation buffer A with BSA (mM): 230 mannitol, 70 sucrose, 10 HEPES, 2 EDTA pH 7.2 with KOH, and 1 mg/mL fatty acid free BSA using a Potter Elvehjem homogenizer (Sigma-Aldrich). Homogenates were centrifuged at 1,300 × g for 3 min at 4°C. The resulting supernatants were collected and centrifuged at 10,000 × g for 10 min at 4°C. The supernatant fraction (F0) was saved for Western blotting. Pellets containing crude mitochondria were overlaid on 30% (v/v) Percoll (Sigma-Aldrich) in buffer B (mM): 250 sucrose, 10 HEPES-Na, 1 EDTA-Na_2_, pH 7.4. Samples were centrifuged (Optima MAX-XP, Beckman Coulter) in a fixed angle rotor at 50,000 × g at 4°C for 45 min. After ultracentrifugation, three fractions were collected and labeled as F1, F2, and F3 from cell samples. Fractions were pelleted at 12,000 × g for 5 min at 4°C for Western blotting. Three independent experiments were conducted and the representative blots were shown.

### Confocal fixed and live-cell imaging

Images were acquired using a Nikon Eclipse T*i* imaging system with a ×100/1.49 Apo TIRF or a x20/0.75 Plan Apo objective, a spinning disk confocal unit (Yokogowa CSU-X1) with 486, 561, and 647-nm diode-pumped solid state lasers, and an ORCA-Flash 4.0 Hamamatsu camera (C11440), controlled by NIS Elements software. Live cell imaging was carried out in transiently transfected HeLa cells as previously described (Zhang et al., 2014). Glass-bottomed dishes were coated with human fibronectin (10 μg/ml, Corning) and 0.1% gelatin (Sigma-Aldrich). Cells were grown on these dishes at 37°C in a humidified atmosphere of 5% CO_2_ in fully supplemented media. Cells were cotransfected with fluorescently tagged GJA1 isoforms and mito-BFP using Lipofectamine 2000 according to manufacture's instructions (Thermo Fisher Scientific). At 16–24 h post transfection, cells were immersed in imaging solution containing HBSS supplemented with 10% FBS, and 1x Mycozap-CL. Images were acquired at 37°C using our Nikon Eclipse T*i* imaging system described above. Active mitochondria were labeled by loading cells for 30 min with imaging solution containing 2.5 μM TMRM (Thermo Fisher Scientific). Pearson's coefficient was calculated using the ImageJ JACoP plugin (Bolte and Cordelières, 2006). Three independent experiments were conducted and 5-10 cells were imaged from each experiment.

### H_2_O_2_ treatment and mitochondrial analysis

HeLa cells were plated on coated glass-bottom dishes and transfected with the following plasmids: GST-GFP, GJA1-43k-GFP, and GJA1-20k-GFP. At 24 h post transfection, samples were treated with 300 μM H_2_O_2_ (Sigma-Aldrich) or PBS in fully supplemented medium for 4 h. Cells were fixed in 4% PFA (Electron Microscopy Services) in PBS for 20 min at RT. Immunolabeling: rabbit anti-Tom20 (1:100, Santa Cruz Biotechnology) and chicken anti-GFP (1:500, Abcam). Alexa Fluors (Thermo Fisher Scientific) were used for secondary antibody detection for 1 h at RT. ProLong Gold Antifade containing DAPI was used to mount samples. Using ImageJ (NIH), the peripheral/central fluorescence ratio was determined by dividing peripheral Tom20 signal by that of the perinuclear and Golgi regions. First, each image is background-subtracted using a rolling ball radius of 50 pixels. Thresholding for the inner region of interest (ROI) encompassing the nucleus was set based on 1/6 of the maximal Tom20 intensity of the entire cell. Signal from the nuclear ROI was removed from the cell. The middle ROI (perinuclear/Golgi) boundary was set by expanding the inner ROI by 4 micrometers. Finally, signal density from the remaining outer ROI (periphery) is divided by the sum of that of the inner and middle ROIs to obtain the ratio (Figure S10). Percentages of cells with connected mitochondria were determined by scoring blinded datasets of immunolabeled Tom20 signal from at least 4 experimental replicates.

### Electron microscopy

HeLa cells were transfected with GST-GFP or GJA1-20k-GFP plasmids as described above. Cells were fixed in 2% glutaraldehyde in PBS at RT for 10 min, scraped, and pelleted by centrifugation at 4,200 × g followed by 16,000 × g. Pellets were fixed for 2 additional hours, and then post fixed with 1% osmium tetroxide followed by incubation with 3% uranyl acetate. Samples were dehydrated in ethanol, treated with propylene oxide, embedded in Spurr resin (Electron Microscopy Services), and sectioned using an ultramicrotome (UCT, Leica). Sections were then mounted on EM grids and stained with uranyl acetate and lead citrate. Images were acquired using the JEM1200-EX, JEOL microscope equipped with a digital camera (BioScan 600W, Gatan). For immunogold labeling, cells were fixed in 4% formaldehyde and 0.1% glutaraldehyde in PBS at RT for 10 min, scraped, and pelleted as described above. Cells were fixed overnight at 4°C in 4% formaldehyde and 0.1% glutaraldehyde in PBS and thereafter mixed with 1.5% low melting temperature agarose. Small pieces of agarose with embedded cells were incubated overnight in 1.85 M sucrose/20% PVP-10/50 mM Hepes pH 7.4. Each piece was mounted on an aluminum pin and snap frozen in liquid nitrogen. Ultra-thin sections were prepared using a cryo-ultramicrotome (UC6, Leica) with an F6 cryo-attachment and a Diatome cryoimmuno (35°) diamond knife. The sections were incubated with rabbit anti-GFP primary antibody (1:200; Abcam) at 4°C overnight. The sections were then incubated with secondary antibody conjugated with 10 nm gold particles (1:20; Ted Pella) and imaged as described above. Electron microscopy work was done by the Electron Imaging Center at California NanoSystems Institute, University of California Los Angeles.

### Tracking of mitochondrial movement

Live cell imaging was performed 24 h post transfection of GST-GFP, GJA1-20k-GFP, or GJA1-20k-del6-GFP together with mito-BFP. Fresh imaging solution, containing either 25 μM nocodazole (to disrupt microtubules) or 0.08% DMSO, was added to cells for 45 min prior to image acquisition. Mito-BFP signal was tracked every 5 s for 5 min. To determine mitochondrial velocity and displacement, the MTrackJ plugin (Meijering et al., 2012) for ImageJ (NIH) was used to track individual mitochondrion moving toward the cell tip within a 60 × 60 μm^2^ ROI. Four separate experiments were performed.

### Statistical analysis

All quantitative data were expressed as mean ± SEM and analyzed using Prism 6 software (GraphPad). For comparison between two groups, unpaired two-tail Student's *t*-test was performed. For comparison among three and more groups, one-way ANOVA followed by either Tukey's or Bonferroni's post-hoc test was performed. For comparison between groups with or without H_2_O_2_ treatment, two-way ANOVA followed by Tukey's post-hoc test was performed. A *P* value of less than 0.05 was deemed statistically significant. For tracking data, histograms of mean velocities of each condition were used for nonlinear Gaussian fitting. Single Gaussian fitting generated the following coefficients of determination (R^2^) to assess the goodness of fit: 0.93 (GST control), 0.95 (GST treated with nocodazole), 0.78 (GJA1-20k), and 0.85 (GJA1-20k-del6). Based on these values, data for GJA1-20k and GJA1-20k-del6 were fitted with the sum of two Gaussian distributions to yield *R*^2^-values of 0.91 and 0.97, respectively.

## Results

### GJA1-20k is localized outside of the ER

In addition to the full-length Cx43 protein, endogenous GJA1-20k (see cartoon in Figure S1A) is highly expressed in primary cardiomyocytes and astrocytes, as well as many cell lines including cancer models (Smyth and Shaw, 2013; Salat-Canela et al., 2014; Ul-Hussain et al., 2014). We previously reported that GJA1-20k primarily localizes to the cytoplasm in reticular structures that correspond to ER/Golgi suggesting a role for GJA1-20k in the Cx43 vesicular transport pathway (Smyth and Shaw, 2013). In detailed confocal imaging of immunofluorescence of HeLa cells labeled with exogenous GJA1-20k-GFP, we also found GJA1-20k positive signal throughout the cell (Figure S1B, arrows). As seen in Figure S1C, there is localization of GJA1-20k-GFP (green) with the ER marker protein disulfide-isomerase (PDI, red) (Pearson's coefficient: *r* = 0.722). However, the more intense clusters do not appear to share ER localization. The larger GJA1-20k clusters either overlap with ER (arrowhead) or exist in shapes independent of ER (arrow). These results prompted us to determine the identity of the structures associated with GJA1-20k.

### GJA1-20k delineates the mitochondrial network

**Figure 1:**
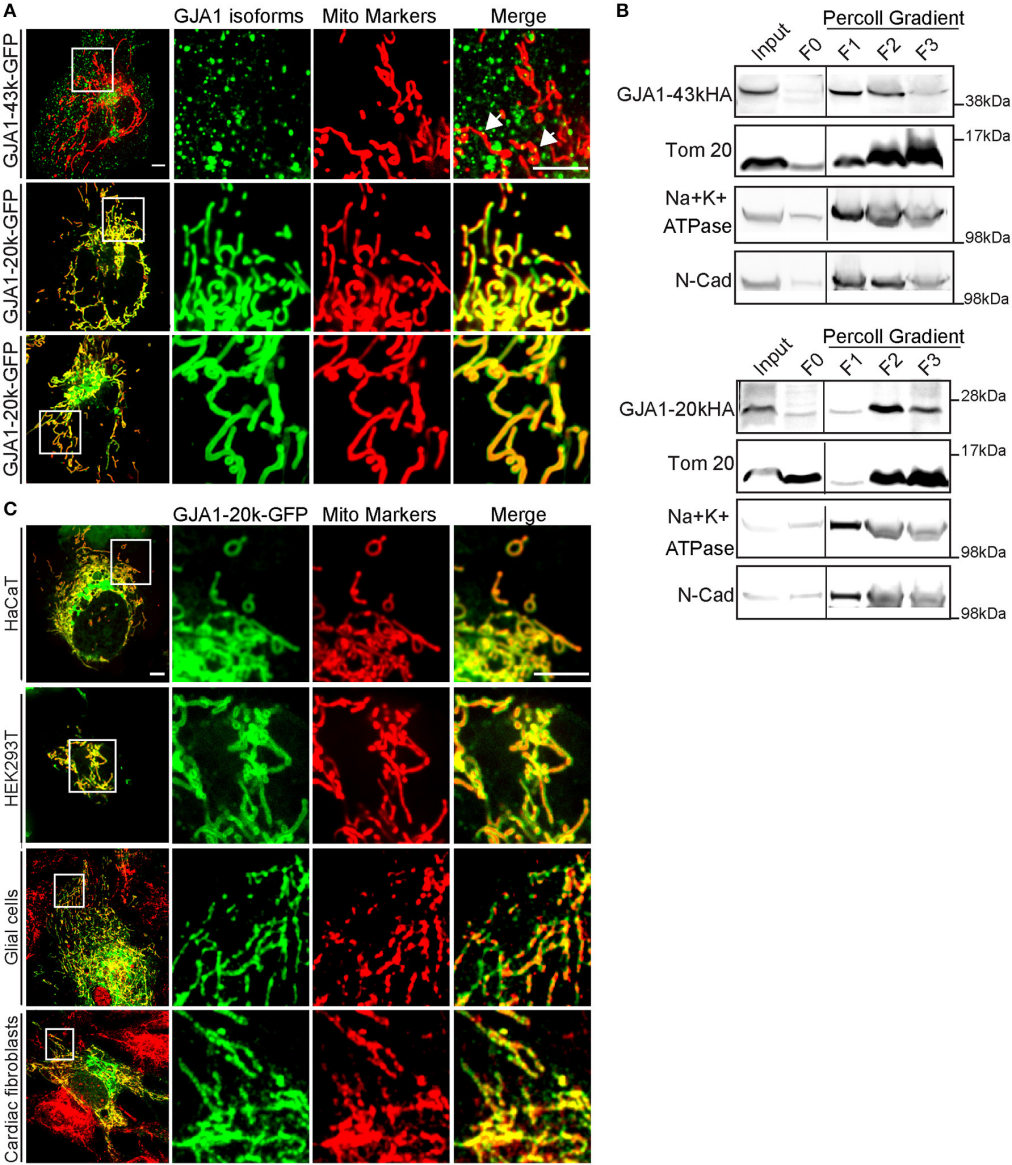
GJA1-20k delineates the mitochondrial network. **(A)** Imaging of live HeLa cells expressing GFP-tagged GJA1-43k or GJA1-20k (green) and mitochondrial markers (red). GJA1-43k associates with mitochondria as small puncta (arrows). GJA1-20k colocalizes with mito-BFP (middle panel) and TMRM (bottom panel). Scale bar = 5 μm. **(B)** Relative enrichment of HA-tagged isoforms in total cell lysate (input), the cytosol supernatant (F0), and membrane fractions of crude mitochondrial pellet (F1–F3). Mitochondria (Tom20). Plasma membrane (Na^+^K^+^ATPase, N-cadherin). Full blots were in Figures S7, S8. **(C)** Live cell imaging of HaCat and HEK293T cells cotransfected with mito-BFP and GJA1-20k-GFP. Immunofluorescence of GFP and Tom20 in primary glial cells and cardiac fibroblasts transfected with GJA1-20k-GFP. Scale bar = 5 μm.

It is recognized that the ER is closely associated with mitochondrial network, participating in dynamic mitochondrial regulation (Friedman et al., 2011). Additionally, accumulating evidence has supported that full length Cx43 is associated with mitochondria (Boengler et al., 2005; Srisakuldee et al., 2014; Gadicherla et al., 2017). Therefore, we decided to examine the association of GJA1-20k with mitochondria. As seen in Figure 1A, while full-length Cx43 (upper panel) (GJA1-43k, with internal start sites mutated to produce only the full-length protein) only partially localizes with the mitochondrial marker mito-BFP (arrows), GJA1-20k strongly co-localizes with mito-BFP (middle panel) as well as with actively respiring mitochondria marked by the membrane potential indicator tetramethylrhodamine methyl ester (TMRM) (bottom panel). GJA1-20k localization is indistinguishable from that of the two mitochondrial markers and appears to function as a mitochondrial marker itself. To address the concern that a GFP tag may interfere with cellular localization of GJA1 isoforms, we took advantage of the previously characterized cervical carcinoma cell line, C33A, that expresses endogenous GJA1-20k (Salat-Canela et al., 2014). Using anti- C-terminal and N-terminal antibodies for Cx43, we can distinguish localization patterns between GJA1-43k (anti- N and C terminal Cx43 antibody positive) and smaller GJA1 isoforms (anti-C terminal Cx43 antibody positive only). As seem in Figure S2, it is primarily the anti C-terminal antibody which outlines mitochondria, indicating that alternatively translated GJA1-20k are preferentially localized to mitochondria, consistent with observation of exogenously expressed GFP-tagged protein. Furthermore, as seen in the same figure, localization of endogenous protein to mitochondria occurs in the peripheral cortex of the cell rather than in more central locations. The imaging data in Figure S2 confirm that even endogenous alternatively translated GJA1-20k preferentially localizes to mitochondria.

We then performed biochemical fractionation to confirm mitochondrial enrichment of GJA1-20k in transfected cells. As seen in Figure 1B, translocase of outer membrane 20 (Tom20) marks mitochondrial fraction and is substantially enriched in fractions F2 and F3. This is concurrent with a corresponding decrease in plasma membrane markers including Na^+^/K^+^-ATPase and N-cadherin. In addition to full length GJA1-43k, GJA1-20k protein is highly enriched in mitochondrial fractions F2 and F3, which supports the mitochondrial localization of GJA1-20k observed in immunofluorescence experiments (Figure 1A). These data indicate that in HeLa cells transfected GJA1-20k has a strong tropism to mitochondria and delineates the entire mitochondrial network.

Since both imaging and biochemical data support enrichment of GJA1-20k in mitochondria, we next sought to confirm that these findings are not restricted to HeLa cells. We obtained human keratinocytes (HaCaT), HEK293 cells, isolated primary neonatal mouse glial cells and cardiac fibroblasts. As seen in Figure 1C, mitochondrial expression of exogenous GJA1-20k is detected in all three types of cells. We have yet to identify cells in which GJA1-20k does not strongly localize to the mitochondrial network.

### GJA1-20k localizes to the interface of mitochondria and microtubules

**Figure 2:**
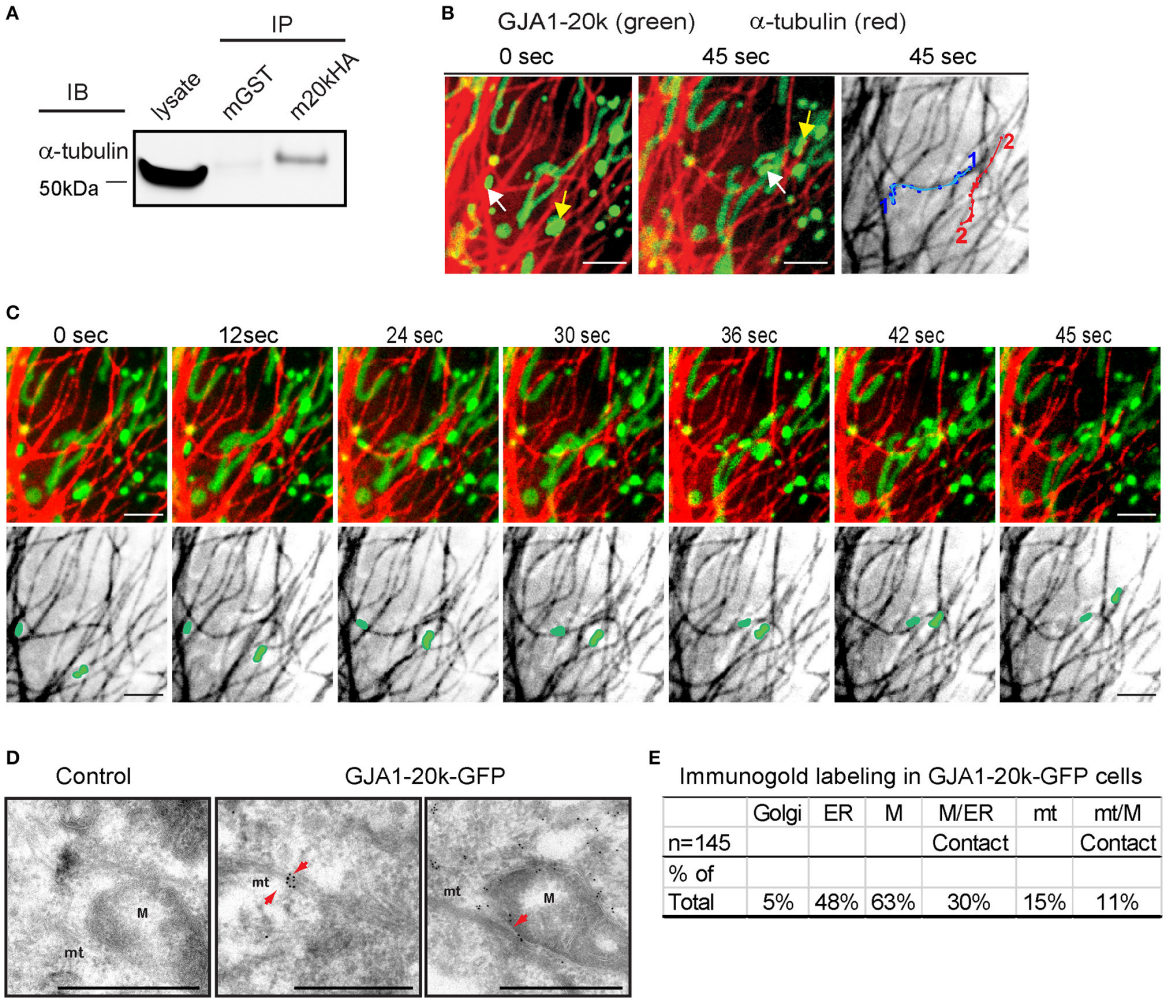
GJA1-20k binds α-tubulin, targeting to the microtubule/mitochondria interface. **(A)** Coimmunoprecipitation between GJA1-20k-HA (IP) and α-tubulin (IB). Antibodies used for IP were labeled as mGST (mouse GST antibody) and m20kHA (mouse HA antibody). Full blot is in Figure S9. **(B)** Live-cell confocal of GJA1-20k-mCherry and α-tubulin-GFP in HeLa cells. Individual GJA1-20k tracks (45 s). **(C)** Time-lapse of merged and tubulin images with moving mitochondria. **(D)** TEM images of GJA1-20k-GFP (immunogold labeled for GFP) occurring along a microtubule bundle, a mitochondrion, and the interface between these structures (arrows) in HeLa cells. Non-transfected negative control (left panel). Microtubules (mt). Mitochondria (M). Scale bar = 500 nm. **(E)** The percent of total images (145) with positive immunogold labeling of each respective organelle is listed in the table. A total of 145 images with gold particles are analyzed.

We have previously reported that GJA1-20k can organize the cytoskeleton trafficking pathways of Cx43 (Smyth and Shaw, 2013; Basheer et al., 2017), which occurs along microtubules (Giepmans et al., 2001; Lauf et al., 2002; Shaw et al., 2007). Microtubule-dependent transport has also been reported for organelles such as mitochondria (Chan, 2006; Wang and Schwarz, 2009; Tanaka et al., 2011). We are interested in the possibility of interaction between GJA1-20k and the microtubule network in respect to mitochondrial transport. As seen in Figure 2A, we found positive co-immunoprecipitation between HA-tagged GJA1-20k and alpha-tubulin, indicating interaction between GJA1-20k and the microtubule highway. Next, using live-cell imaging, we identified that movements of mitochondria containing GJA1-20k signal, when recorded over 45 s, follow trajectories defined by microtubules (Figures 2B,C and Supplemental Video 1), indicating that microtubules might function as guides for GJA1-20k associated mitochondrial movement.

If GJA1-20k facilitates mitochondrial interaction with microtubules, then its localization is expected to be enriched at points of interaction. The confocal live cell imaging clearly demonstrates that mitochondria follow microtubule tracks. However, live cell confocal imaging cannot provide sufficient detail on spatial localization of GJA1-20k GFP due to the resolution of confocal imaging, especially when projecting signals of the three-dimensional organelle to two-dimensional images. Therefore, we used transmission electron microscopy (TEM) together with immunogold labeling of exogenous GJA1-20k to explore in high resolution the spatial relationship among GJA1-20k, mitochondria, and microtubules. Consistent with the confocal imaging results, mitochondria in GJA1-20k-expressing cells are closely associated with microtubules, stretching along them (Figure 2D, and Figure S3). Moreover, immunogold labeling further confirmed that GJA1-20k targets to mitochondria. More than 60% of total immunogold positive images contain gold particle-labeled GJA1-20k in mitochondria (Figure 2E), which is further enriched at mitochondrial membrane. Not surprisingly, cellular organelles such as ER and Golgi were also identified by immunogold labeling in GJA1-20k transfected cells. Although only 15% (22) of all 145 images contain gold particle-labeled GJA1-20k at microtubules, the majority (16/22, 73%) of them are associated with mitochondria at the microtubule interface, which is within 0.1 micron of microtubules (Figures 2D,E). It appears that GJA1-20k helps to load the mitochondrion along the microtubule for transport. Together, the data in Figure 2 provide biochemical, live-cell imaging, and TEM evidence in support of a role for GJA1-20k in microtubule-based mitochondrial transport.

### GJA1-20k mediates microtubule-based mitochondrial transport

GJA1-20k, beginning with amino acid 213, contains a portion of the fourth transmembrane domain of Cx43, and the entire cytoplasmic C-terminal tail (Figure S1A). Like its full-length counterpart, this 170 amino acid isoform includes the microtubule-binding domain (MTBD), which occurs at amino acids 234–259 of Cx43 (Giepmans et al., 2001; Saidi Brikci-Nigassa et al., 2012) (Figure S3). Six residues (^239^RV^240^ and ^247^YHAT^250^) in MTBD have been found critical for microtubule/Cx43 interaction (Saidi Brikci-Nigassa et al., 2012).

**Figure 3:**
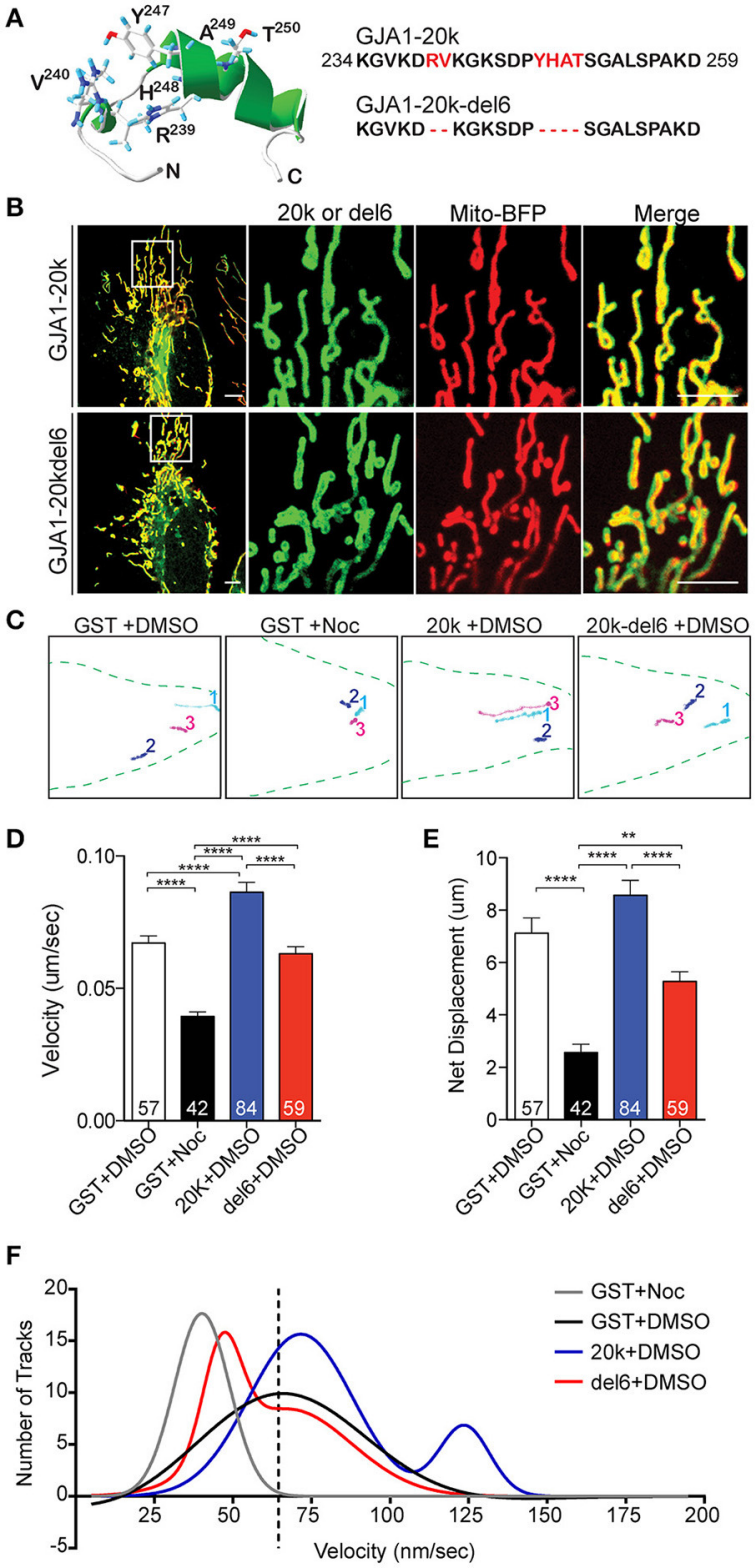
Key microtubule-interacting residues mediate trafficking. **(A)** Swiss PDB Viewer rendering of the MTBD. Six residues (red) were removed in GJA1-20k-del6. **(B)** Live-cell images of GJA1-20k vs. GJA1-20k-del6 (green). Mito-BFP (red). Scale bar = 5 μm. Noc = nocodazole. **(C)** Live-cell mitochondrial tracks (mito-BFP) in each group. **(D–E)** Tracked speed and displacement, respectively. Data are mean ± SEM (*n* = 4 experimental repeats, 10–30 tracked mitochondria from 5 to 10 cells from each repeat were included. N in each column is number of mitochondria tracked), ^**^p < 0.01, ^****^p < 0.0001, one-way ANOVA, Tukey's *post hoc* test. **(F)** Nonlinear Gaussian fitting of track velocity frequency and distribution. The *R*^2^ coefficient of Gaussian fitting are: 0.93 (GST, single Gaussian fitting), 0.95 (GST+Noc, single Gaussian fitting), 0.91 (20 k, double Gaussian fitting), 0.97 (del6, double Gaussian fitting).

Based on the association between GJA1-20k with mitochondria (Figures 1, 2) and microtubules (Figure 2), we tested whether GJA1-20k mediates microtubule-based mitochondrial transport. We generated a mutant lacking the six residues essential for microtubule interaction (GJA1-20k-del6) (Figure 3A; Figure S11). GJA1-20k and mutated GJA1-20k-del6 were overexpressed in HeLa cells. Interestingly, as seen in Figure 3B, GJA1-20k-del6 retains a mitochondrial localization which is indistinguishable from wildtype GJA1-20k, suggesting that the MTBD is not necessary for GJA1-20k localization to mitochondria.

We next examined how the mutation of MTBD affects dynamics of mitochondrial transport in transfected cells. Mitochondrial movements were assessed by tracking individual mitochondrion over a 5-min period. Representative tracks are presented in Figure 3C, with original movies in Supplemental Videos S2–S5. Mitochondria were tracked in the presence of GJA1-20k, GJA1-20k-del6, or the microtubule disruptor nocodazole (noc). Quantitative analysis (Figures 3D,E) revealed that nocodazole significantly reduces average mitochondrial transport speed and net displacement relative to DMSO treated control cells. In contrast, GJA1-20k increases average mitochondrial speed, while average mitochondrial speed and displacement in GJA1-20k-del6 transfected cells were not significantly different from control. Frequency distribution analyses of individual average mitochondrial speeds (per 5 min windows) were fit with single Gaussian curves (single Gaussian unless *R*^2^ fit was less than 0.9 and then double Gaussian curves were used to fit the data, Figure 3F). The original histograms on which the curve fits are based have been removed for clarity in Figure 3F. The interested reader is referred to Figure S4 for the original data. The primary peaks of GST (black), GJA1-20k (blue), and GJA1-20k-del6 (red) curves were similar at an average speed of ~65 nm/s. Nocodazole shifted the GST curve to the left (gray), indicating a loss of fast mitochondrial movement on microtubules, whereas a secondary curve to the right (movement at higher speeds) occurred only with GJA1-20k cells (blue) and the area under the curve indicates about 24% population of mitochondria are traveling at the higher speed. These data indicate that in cells with exogenous GJA1-20k containing an intact MTBD, a subpopulation of mitochondria travels at twice the median speed, which is consistent with kinesin-based transport (Wang and Schwarz, 2009; Tanaka et al., 2011). In contrast, the secondary peak of GJA1-20k-del6 cells is shifted to the left (lower speeds, red), closer to the nocodazole peak, indicating loss of mitochondrial transport on microtubules. This is further supported by reduced tubulin interaction with GJA1-20k-del6 (Figure S11). Thus, we conclude that exogenous GJA1-20k increases the instances of mitochondria utilizing microtubule-based transport. The rapid outward-directed movement supports the role of GJA1-20k in increasing microtubule-based transport of mitochondria to the cell periphery.

### GJA1-20k preserves mitochondrial distribution during stress

**Figure 4:**
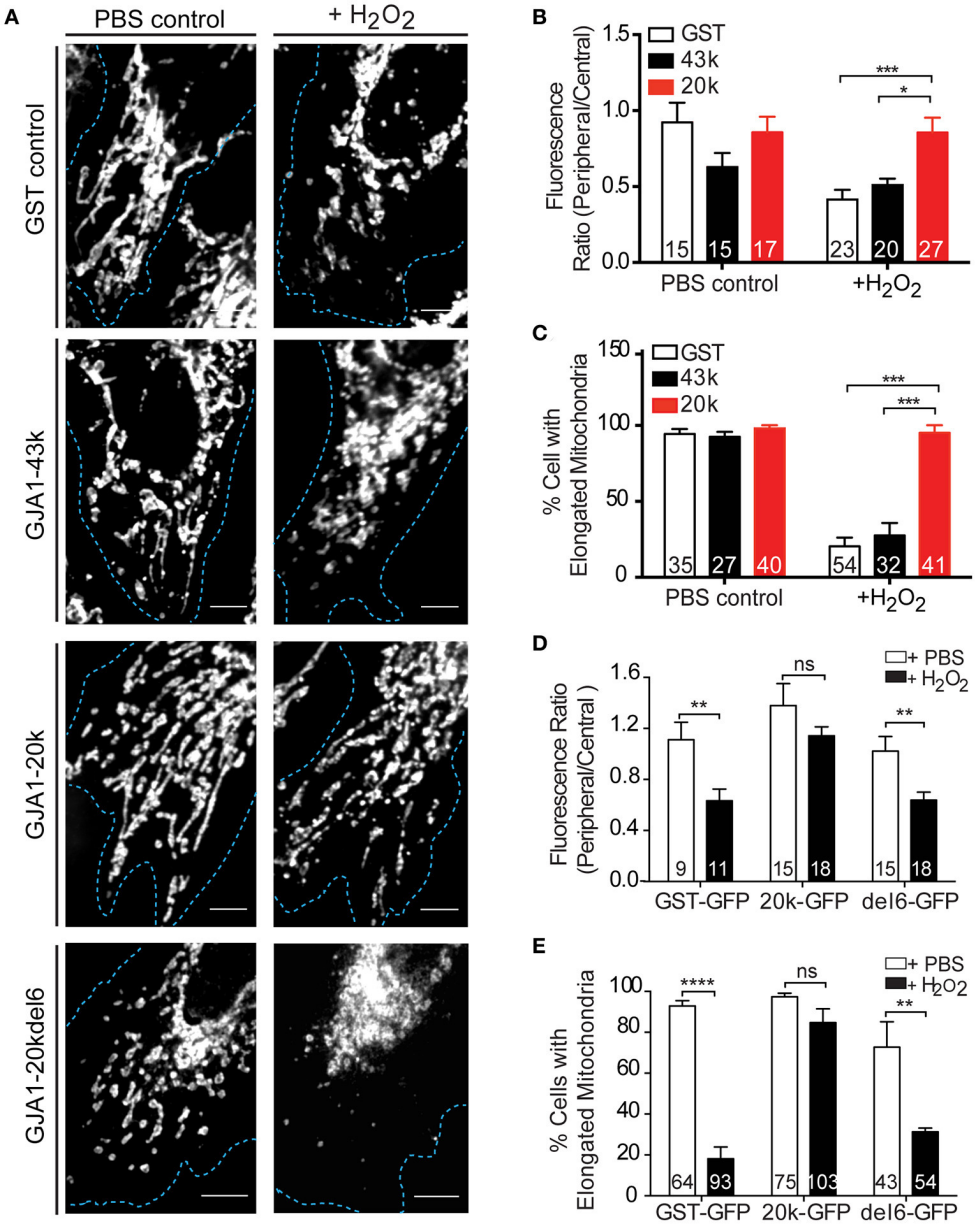
GJA1-20k limits mitochondrial fragmentation upon oxidative stress. **(A)** Mitochondria morphology and distribution (Tom20) in HeLa cells expressing GFP-tagged GST, GJA1-43k, or GJA1-20k, or GJA1-20kdel6 treated with PBS or 300 μM H_2_O_2_. Cell borders (blue). Scale bar = 5 μm. See also Figure S6. **(B)** Periphery versus cell center Tom20 fluorescence intensity and **(C)** percentage of cells containing elongated mitochondria comparing GFP-tagged GST, GJA1-43k and GJA1-20k. **(D)** Peripheral vs. central mitochondria (Tom20) (See Figure S10) and **(E)** percentage of cells with elongated mitochondria comparing GFP-tagged GST, 20k, 20k-del6 in PBS or H_2_O_2_. All data are mean ± SEM (*n* = 4 repeats), ^*^p < 0.05, ^**^p < 0.01, ^***^p < 0.001, ^****^p < 0.0001, two-way ANOVA, Tukey's *post-hoc*-test.

Since our results indicate that GJA1-20k mediates mitochondrial movement along microtubules (Figures 2, 3), we next asked whether GJA1-20k can safeguard a distributed mitochondrial network during cellular stress. Mitochondrial morphology in fixed cells, as determined by Tom20 immunofluorescence, was observed in HeLa cells subjected to PBS or 300 μM H_2_O_2_ for 4 h. Confocal imaging of cells expressing GFP-tagged GST, GJA1-43k, or GJA1-20k revealed a distributed mitochondrial network in all groups treated with PBS (Figure 4A, left panels and Figure S5). Upon H_2_O_2_ treatment however, mitochondria became centralized and fragmented in cells expressing GST and GJA1-43k, indicating a damaged mitochondrial network. Interestingly, the majority of GJA1-20k transfected cells retained well distributed network and elongated mitochondrial morphology (Figure 4A, right panels). We quantified mitochondrial localization as a ratio of peripheral to central distribution and observed that GJA1-20k significantly rescued mitochondrial peripheralization during H_2_O_2_ stress (Figure 4B). We then quantified elongated mitochondria and found that GJA1-20k rescued mitochondrial morphology as well, when cells were treated with H_2_O_2_ (Figure 4C). These data indicate that introduction of GJA1-20k maintains peripheral mitochondria, limiting organelle network collapse when subjected to metabolic stress. It is possible that GJA1-20k could confer a protective and pro-survival effect through its regulation of mitochondrial distribution and morphology.

In Figure 3 we introduced the GJA1-20k-del6 mutant which lacks the key residues in the MTBD of GJA1-20k, and observed persistent mitochondrial localization of GJA1-20k-del6 but impaired microtubule transport (Figures 3B,F). We tested whether GJA1-20k-del6 could rescue mitochondrial localization and morphology when cells are subjected to H_2_O_2_ stress. The results, in Figures 4A,D,E are negative. Apparently without the microtubule binding capacity, GJA1-20k-del6 loses the capability of GJA1-20k to rescue mitochondrial network integrity during stress, indicating the importance of microtubule based transport for mitochondrial response to oxidative stress.

## Discussion

**Figure 5:**
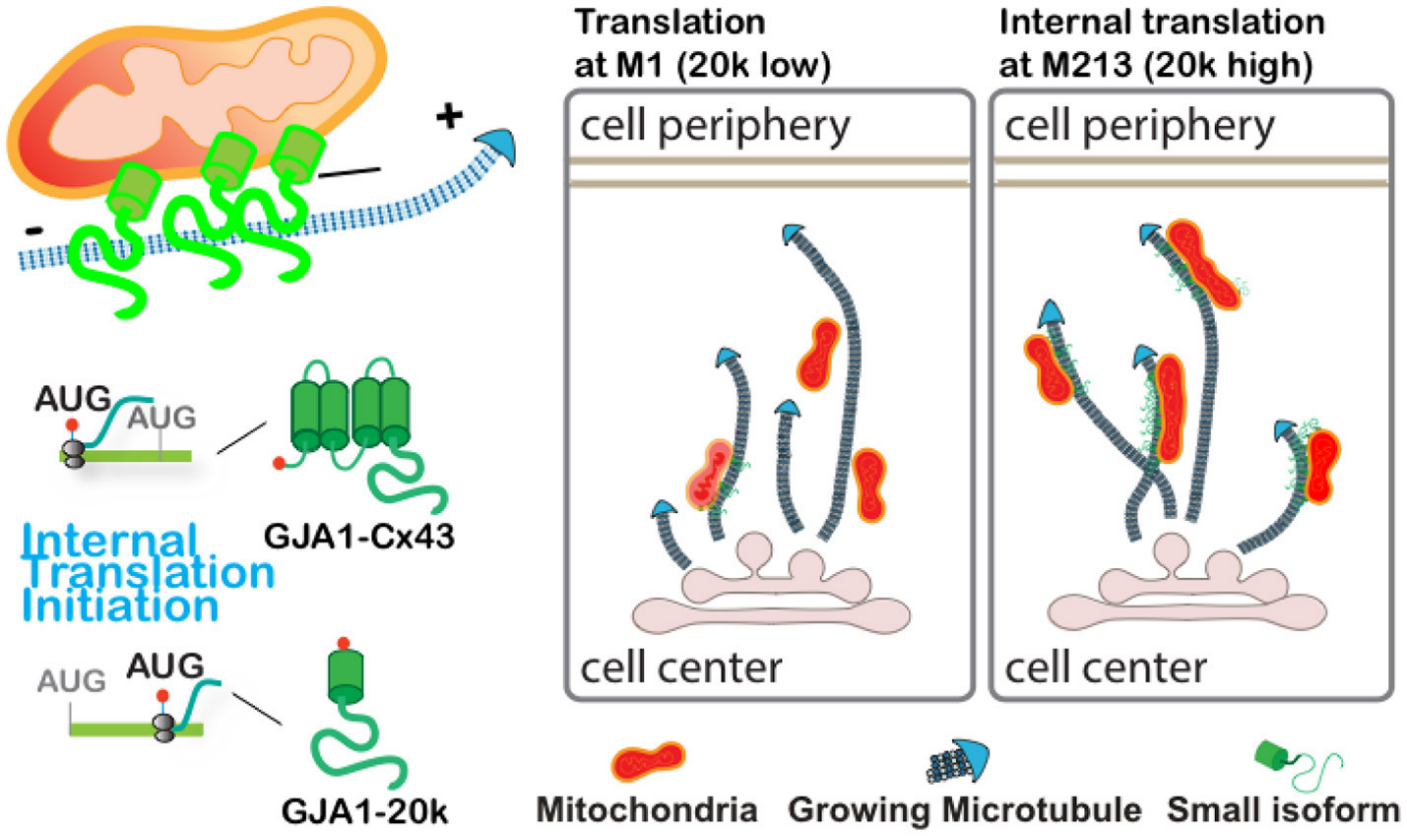
Graphical illustration demonstrates that GJA1-20k localizes to mitochondria and facilitates microtubule-dependent mitochondrial transport to cell periphery and limits mitochondrial fragmentation upon oxidative stress.

The present study identified that GJA1-20k, produced via alternative translation from the same mRNA encoding gap junction protein Cx43, preferentially localizes to mitochondria and mitochondria/microtubule interfaces. We find that GJA1-20k facilitates microtubule-dependent trafficking of mitochondria to cell periphery, thereby preserving distributed mitochondrial network and preventing mitochondrial fragmentation induced by oxidative stress (depicted in the cartoon in Figure 5). This work reveals a novel function of GJA1-20k in regulating mitochondrial distribution in response to cellular stress.

### Alternative translation of Cx43 and its functional significance

It is commonly accepted that, in eukaryotic cells, mature mRNA molecules encode and correspond to single protein products. In recent years, accumulating evidence has suggested that alternative translation start sites are highly conserved in the mammalian genome and that proteome diversity produced via alternative translation may be more important than previously thought (Kochetov, 2008; Bazykin and Kochetov, 2011; de Klerk and ‘t Hoen, 2015). Cx43 is the first mammalian ion channel identified to undergo alternative translation (Smyth and Shaw, 2013; Salat-Canela et al., 2014; Ul-Hussain et al., 2014). Up to six alternatively translated Cx43 isoforms are produced from *GJA1* mRNA in the heart (Smyth and Shaw, 2013), all of which are N-terminal truncated and contain either the entire or distal portion of the C-terminal tail. The loss of one or more transmembrane domains of full length Cx43 gives rise to different protein solubility which can result in different cellular distribution, biophysical properties and their functions. In addition to its role as a chaperone to facilitate full-length GJA1-43k protein trafficking to the cell-cell border (Smyth and Shaw, 2013; Basheer et al., 2017), we now report that by interacting with microtubules GJA1-20k also aids trafficking and distribution of mitochondrial organelles (Figures 2, 3), resulting in cellular protection from stress (Figure 4). With the microtubule binding domain located at proximal C-terminus, it is likely that the N-terminal partial fourth transmembrane domain of GJA1-20k can interact with mitochondrial membrane lipids and/or other mitochondrial adaptor proteins to mediate microtubule dependent interaction and transport of mitochondria.

Alternative translation is a highly regulated process. In this study, we took advantage of HeLa cell lines with little endogenous expression of Cx43 which provides a clear background to study function of alternatively translated smaller isoforms. However, it is known that cellular stress may act as a trigger for alternative translation to generate isoforms that increase survival. For instance, the protein level of GJA1-20k in mouse primary astrocytes nearly doubled following exposure to intermittent hypoxia for 120 min (Ul-Hussain et al., 2014). Similarly, signaling pathways involved in survival, such as mTOR and Mnk1/2 have been linked to upregulation of GJA1-20k by between 2- and 50-fold (Smyth and Shaw, 2013; Salat-Canela et al., 2014). Therefore, our study, though in a heterologous expression system, provides a glimpse of functional impact of GJA1-20k. Given the dynamic regulation of alternative translation, understanding the function of smaller isoforms of Cx43 provides a rare opportunity to identify novel protein targets required for cell survival.

### Microtubule-based mitochondrial transport and distribution

Mitochondria form an extensively regulated and highly dynamic organelle network that is responsible for energy production, calcium homeostasis, cell signaling, cell growth and survival (Frederick and Shaw, 2007). Highly regulated intracellular movement of mitochondria and their appropriate distribution are crucial for the function and integrity of the overall network (Chan, 2006). Spatial mobility is one of the primary means by which the mitochondria can alter their function to adapt to environmental changes and cellular stresses.

In multicellular eukaryotes, mitochondria move along microtubule tracks by attaching to motors such as kinesin for anterograde transport and dynein for retrograde transport (Hollenbeck and Saxton, 2005; Russo et al., 2009). Impaired mitochondrial movement and the resulting aberrant distribution are detrimental for the cell function, which is particularly manifest in highly polarized cells (Frederick and Shaw, 2007; Saotome et al., 2008). Here we find that, even in HeLa cells, pharmaceutical disruption of microtubules dramatically reduces mitochondrial transport (Figure 3), confirming the role of microtubules in mitochondrial movement. The alternatively translated small isoform of Cx43, GJA1-20k, when expressed exogenously, facilitates mitochondrial trafficking along microtubules. Our finding demonstrates that GJA1-20k not only regulates microtubule dependent vesicular protein cargo trafficking (Smyth and Shaw, 2013; Basheer et al., 2017), but also microtubule dependent organelle transport (Figures 3, 4). A mutant of GJA1-20k (GJA1-20k-del6) which lacks ability to bind microtubules still reaches mitochondrial membrane, suggesting microtubule transport is not needed for mitochondrial affinity and possibly GJA1-20k-del6 may localize to mitochondria at earlier stages of mitogenesis. However GJA1-20k-del6 fails to rescue mitochondrial mobility and to maintain mitochondrial network distribution in presence of H_2_O_2_ (Figure 4). Through its interaction with microtubules, GJA1-20k may act as an adaptor protein to facilitate loading of mitochondria onto microtubule trafficking highways for movement and distribution within the cell. In future studies, it will be important to address whether GJA1-20k also interacts with known microtubule-based motor/adaptor transport complex proteins (Chan, 2006; Wang and Schwarz, 2009; Tanaka et al., 2011) which regulate mitochondrial trafficking velocity and directionality.

Of note and in additional to spatial mobility, mitochondria engage in repeating cycles of fusion and fission to intermix membrane lipids and matrix contents to ensure organelle network health and function (Ong et al., 2010, 2013; Huang et al., 2013). Maintenance of an intact network of elongated mitochondria protects against mitophagy and cell death through evasion of autophagosome engulfment (Gomes et al., 2011; Rambold et al., 2011). To buffer against damage and dysfunction, impaired mitochondria undergo fission and are distributed throughout the remaining healthy network (Nakada et al., 2001; Ono et al., 2001). In all cells, there exists a dynamic equilibrium between mitochondrial fission and fusion. Both fission and fusion are carried out by a distinct group of proteins, including Drp1, FIS1, OPA1, and mitofusin 1/2. It is possible that, through interaction with one or more of these proteins, GJA1-20k tips the balance between fission and fusion in favor of elongated morphology of mitochondria.

## Conclusions

We have identified that GJA1-20k enriches at mitochondria and facilitates microtubule-based mitochondrial transport which preserves organelle network integrity upon oxidative stress. GJA1-20k, a protein that was only recently recognized to exist by alternative translation (Smyth and Shaw, 2013; Salat-Canela et al., 2014; Ul-Hussain et al., 2014), provides a means by which otherwise traditional Cx43 gap junction protein can have a highly diverse repertoire of non-canonical roles. It is possible that the GJA1-20k peptide could be developed into a therapeutic to prevent mitochondrial fragmentation in situations of cellular stress.

## Author contributions

YF, S-SZ, and SX contribute equally to the experimental design, acquisition, interpretation of data and drafting the manuscript. WB, RB, and IE contribute to experimental design, data acquisition and drafting the manuscript. TH and RS contributed to all aspects of this work. All authors agree to be accountable for accuracy and integrity of the work.

### Conflict of interest statement

The authors declare that the research was conducted in the absence of any commercial or financial relationships that could be construed as a potential conflict of interest.

## Abbreviations

mRNA: messenger RNA
C-terminal: carboxyl-terminal
ER: endoplasmic reticulum
PDI: protein disulfide-isomerase
TMRM: indicator tetramethylrhodamine methyl ester
Tom20: translocase of outer membrane 20
TEM: transmission electron microscopy
NMR: nuclear magnetic resonance
MTBD: microtubule-binding domain
H_2_O_2_: hydrogen peroxide
Noc: nocodazole
DMSO: dimethyl sulfoxide.
