# Supplementary figures and images for: Cx43 Isoform GJA1-20k Promotes Microtubule Dependent Mitochondrial Transport
Source: Front Physiol. 2017 Nov 7;8:905. doi: 10.3389/fphys.2017.00905 (PMC5682029; doi:10.3389/fphys.2017.00905)

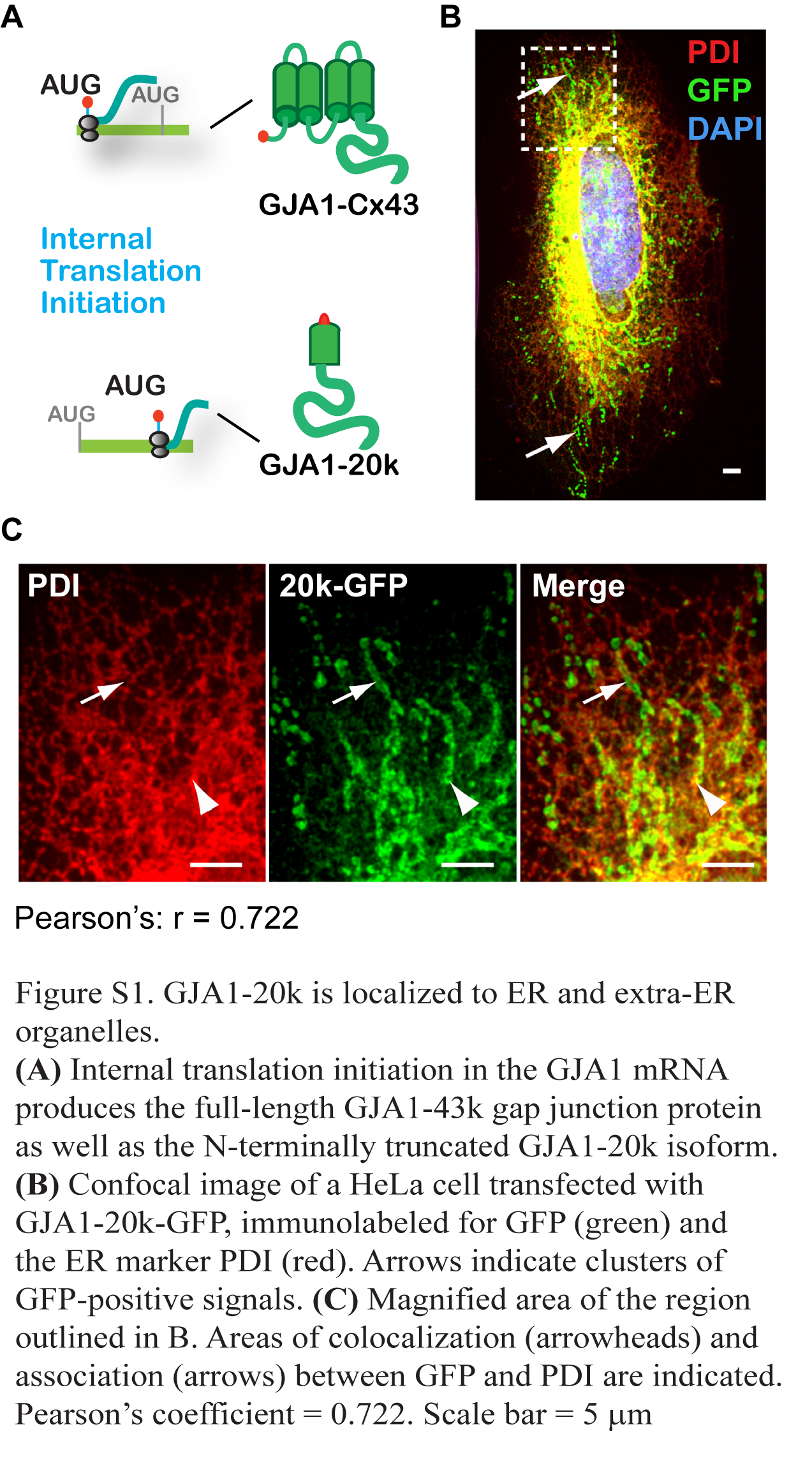

Supplement: Supplementary file 1 [file Image1.TIF]

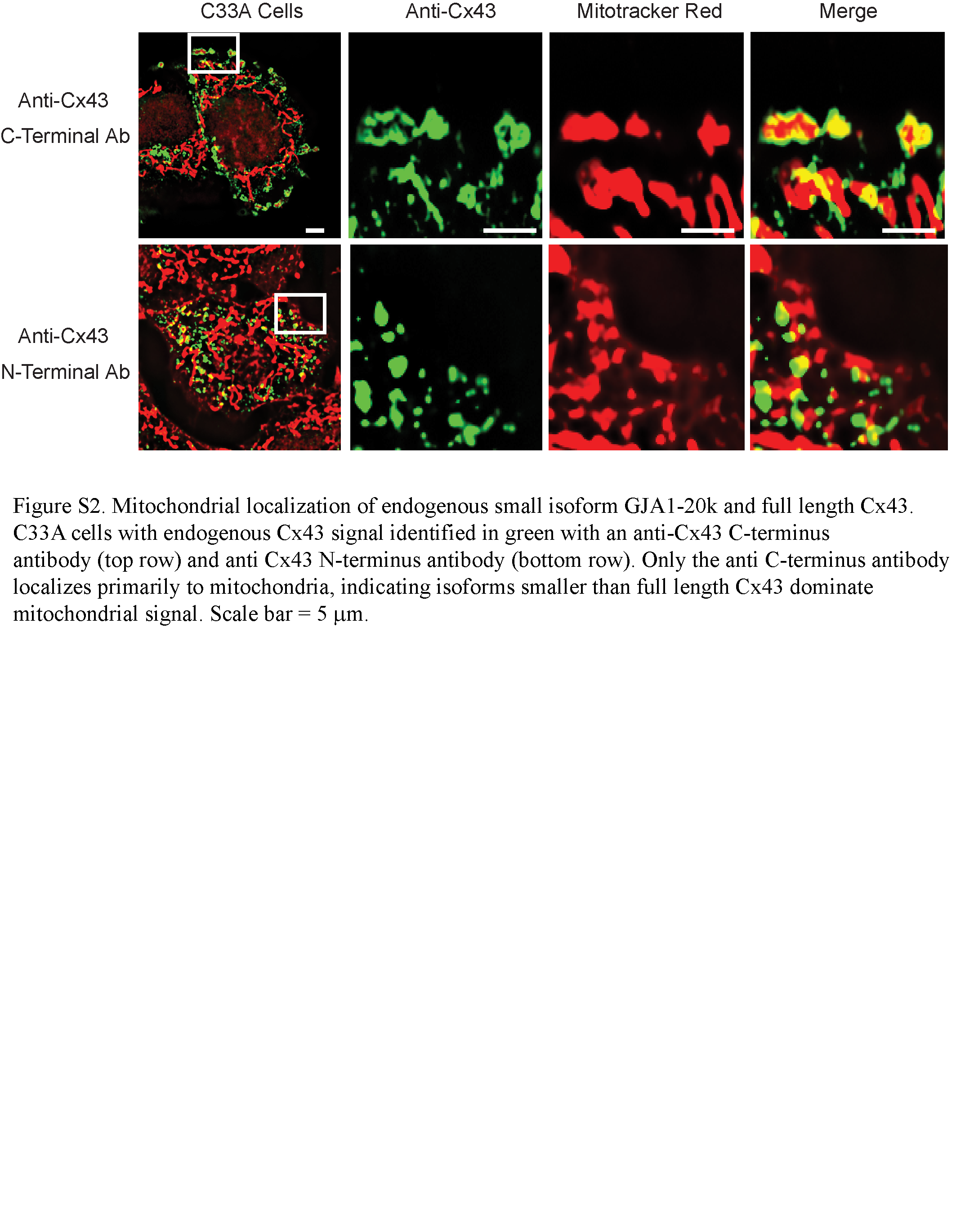

Supplement: Supplementary file 2 [file Image2.TIF]

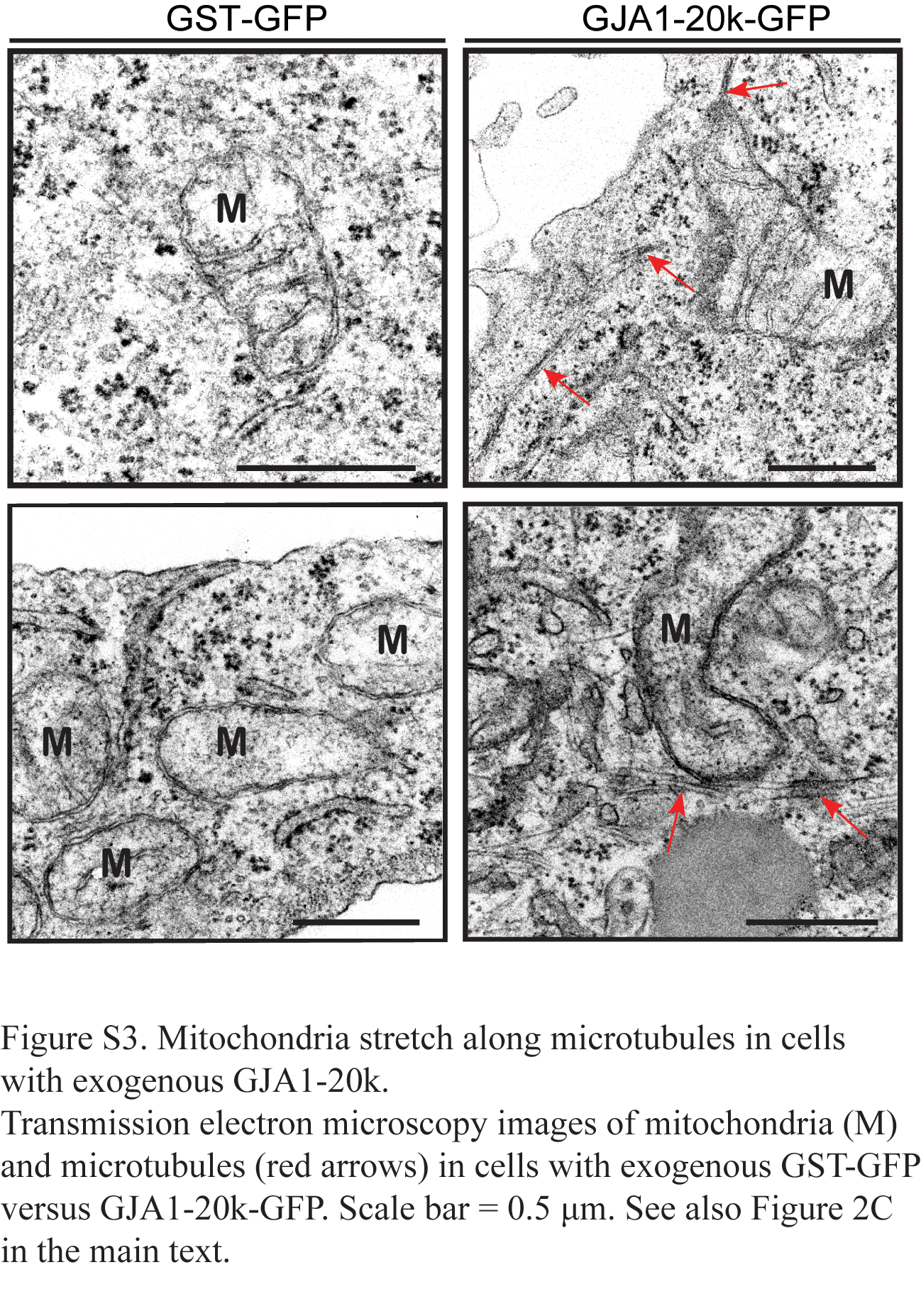

Supplement: Supplementary file 3 [file Image3.TIF]

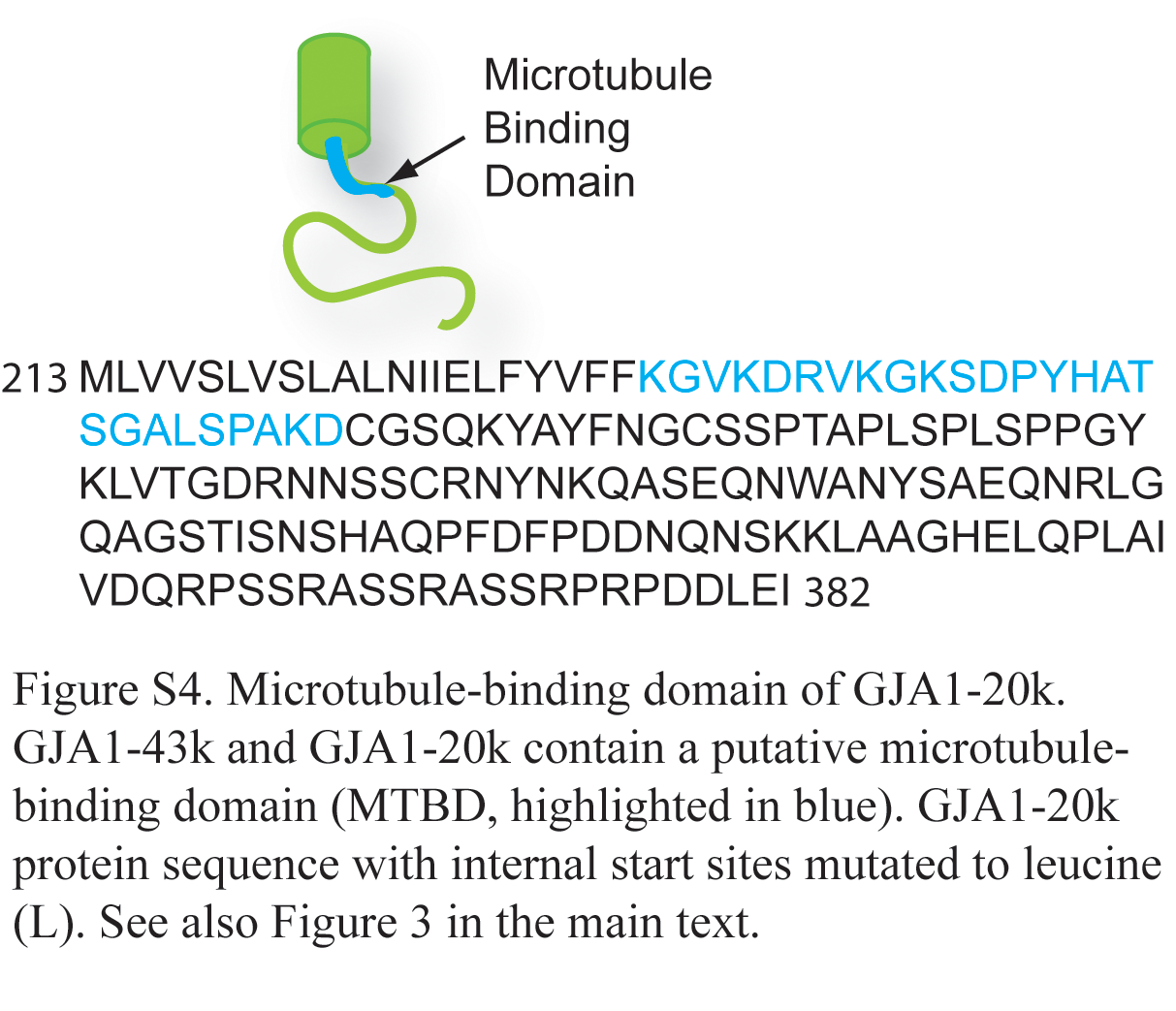

Supplement: Supplementary file 4 [file Image4.TIF]

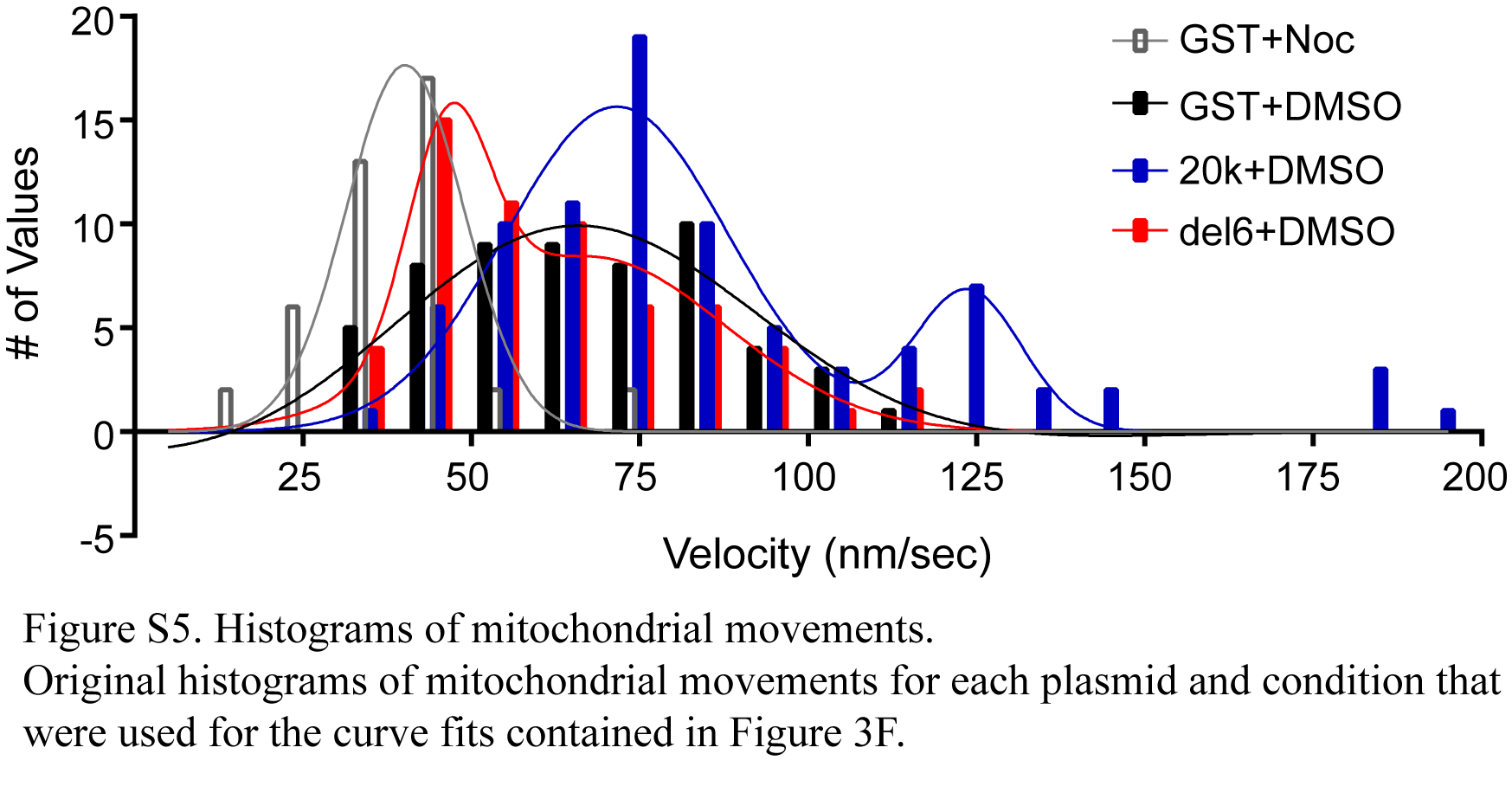

Supplement: Supplementary file 5 [file Image5.TIF]

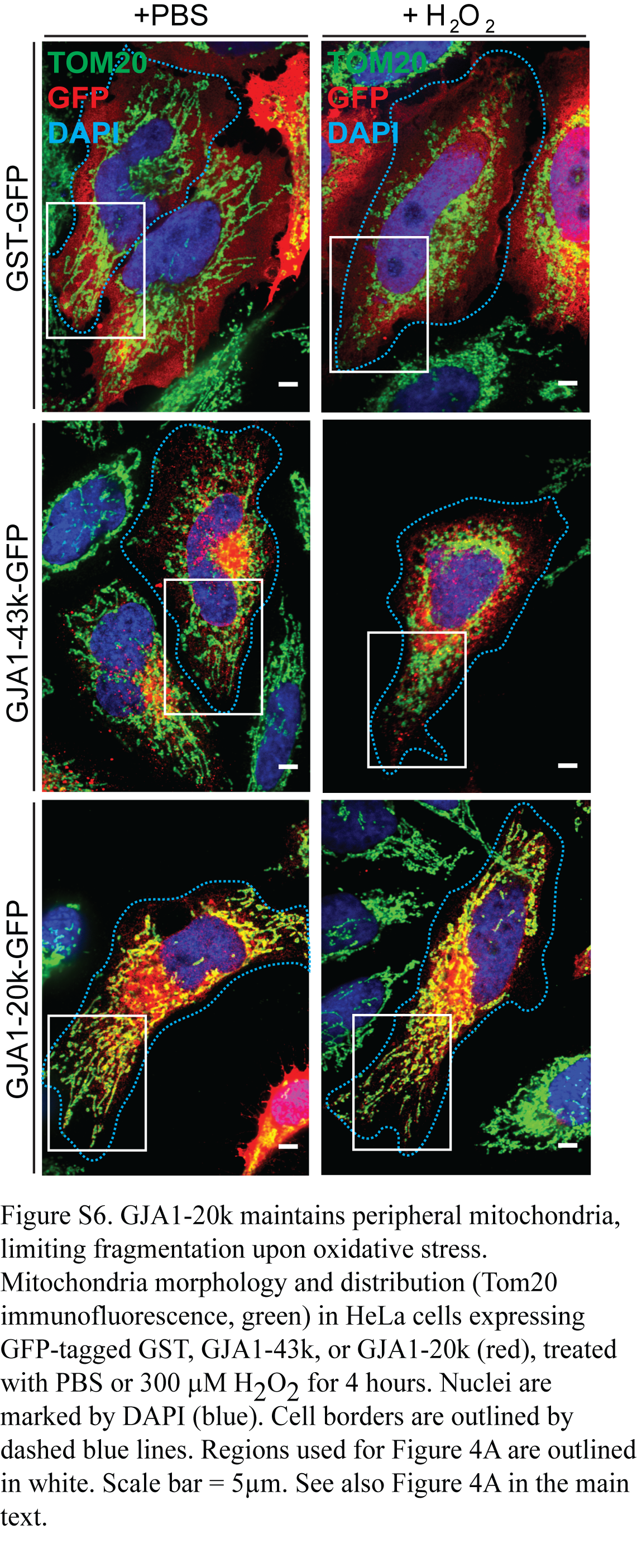

Supplement: Supplementary file 6 [file Image6.TIF]

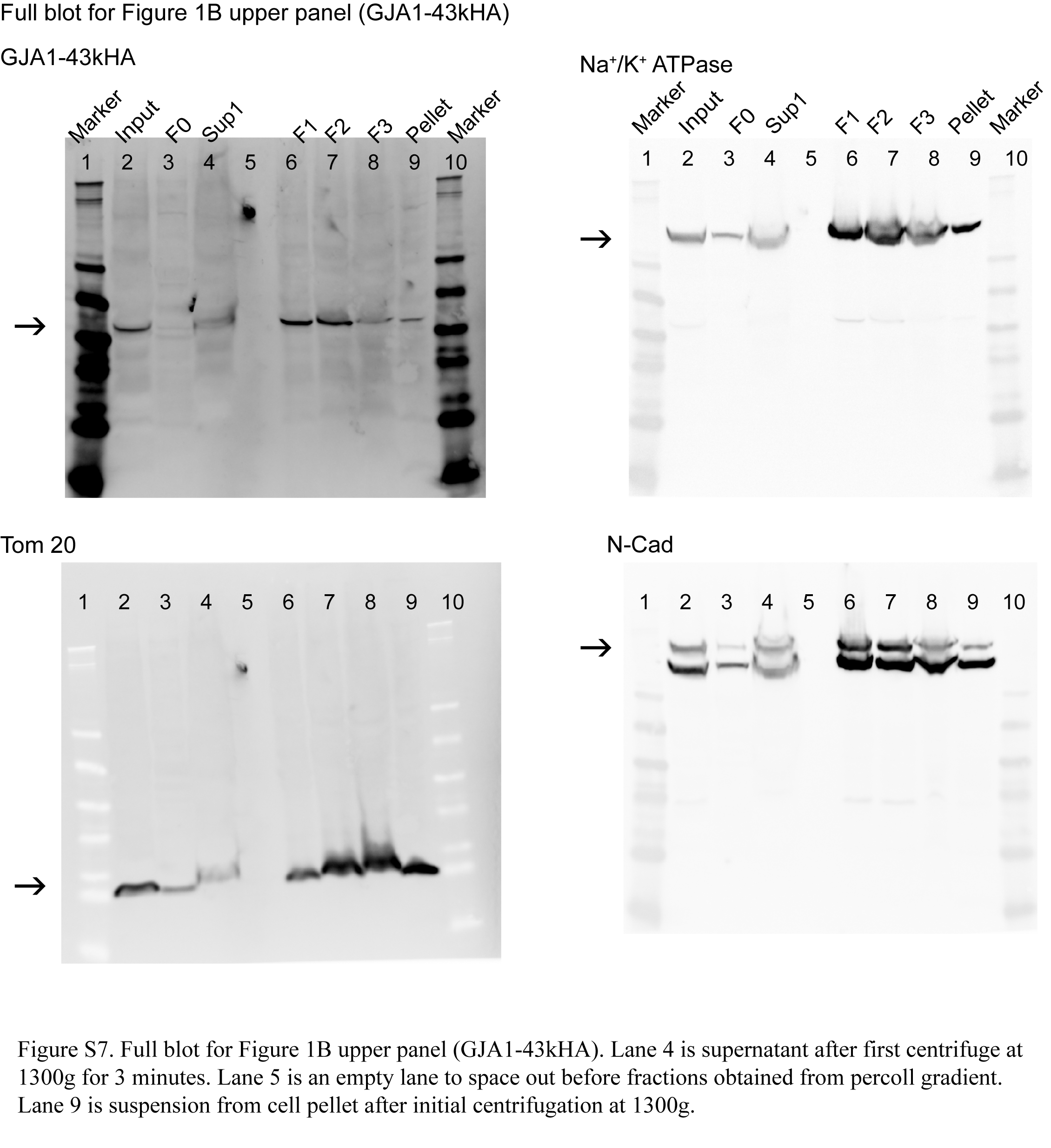

Supplement: Supplementary file 7 [file Image7.TIF]

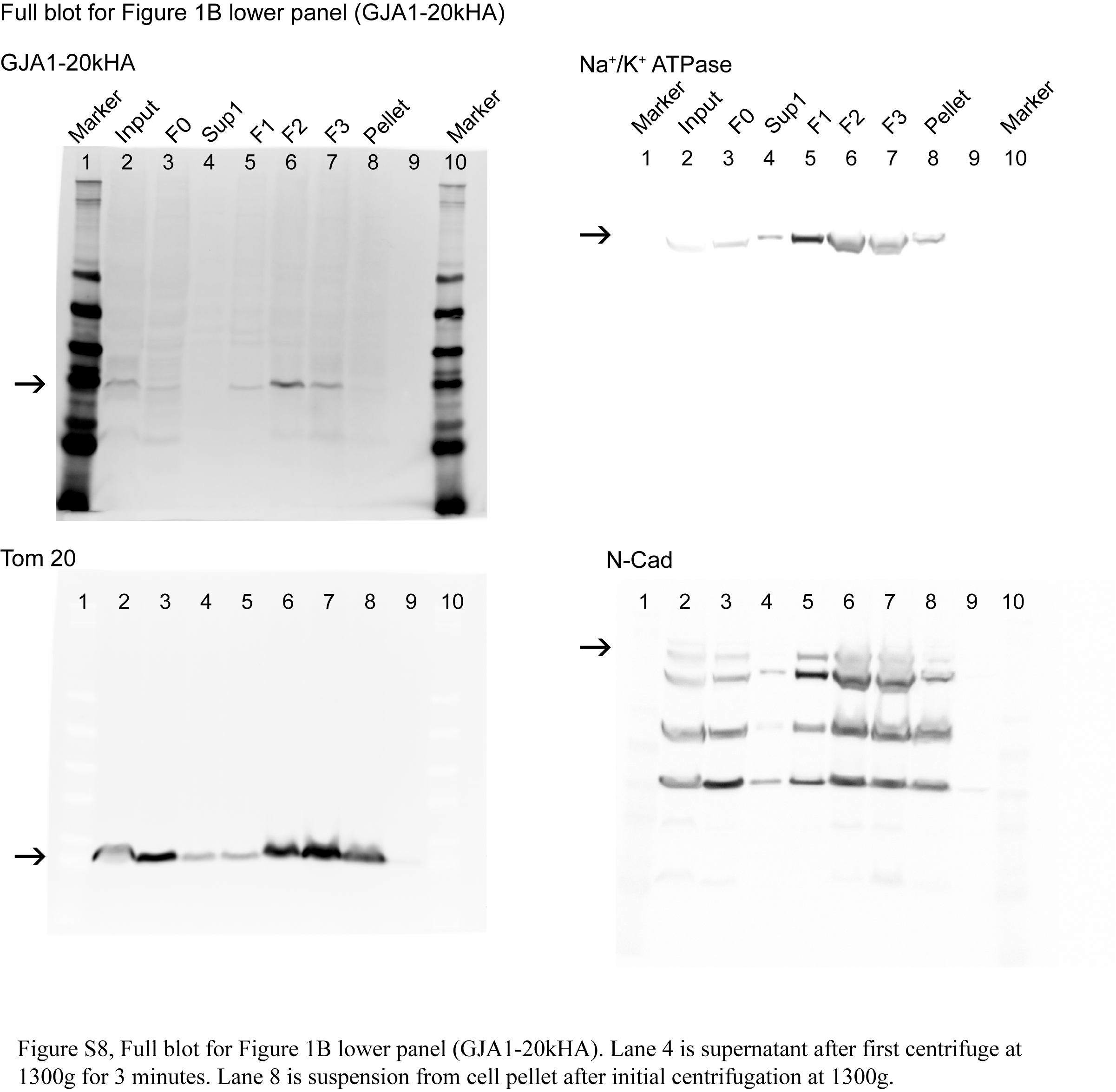

Supplement: Supplementary file 8 [file Image8.TIF]

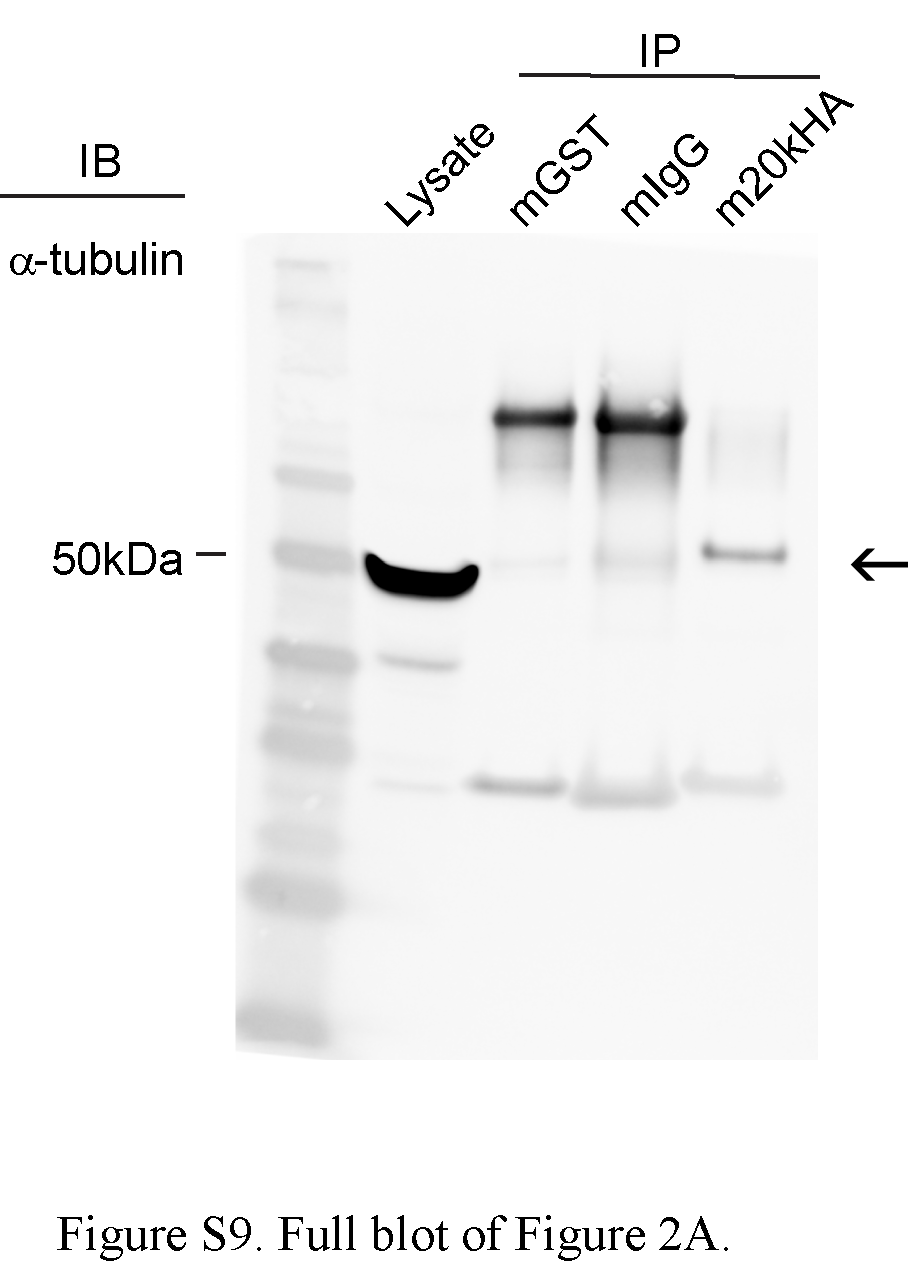

Supplement: Supplementary file 9 [file Image9.TIF]

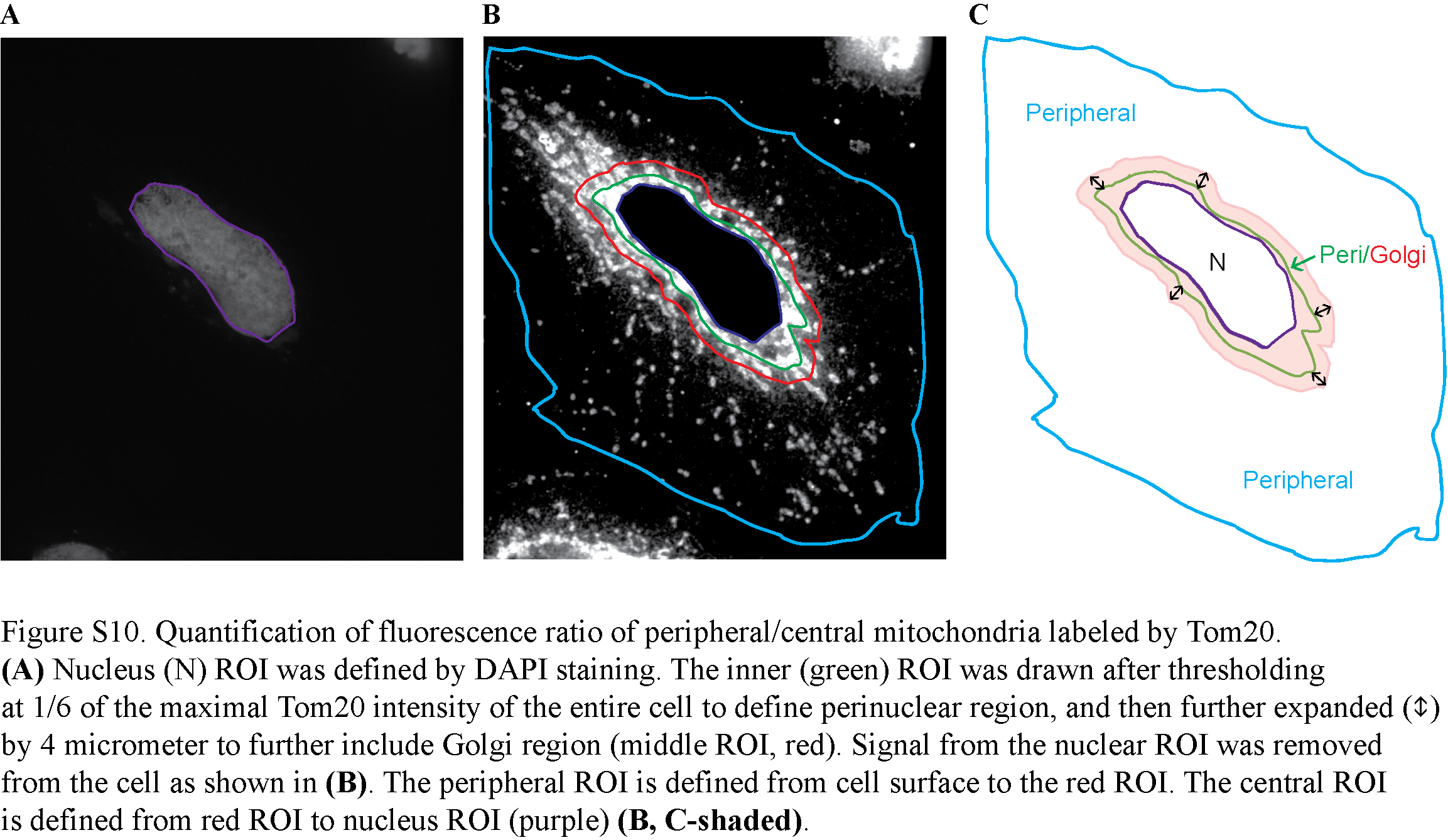

Supplement: Supplementary file 10 [file Image10.TIF]

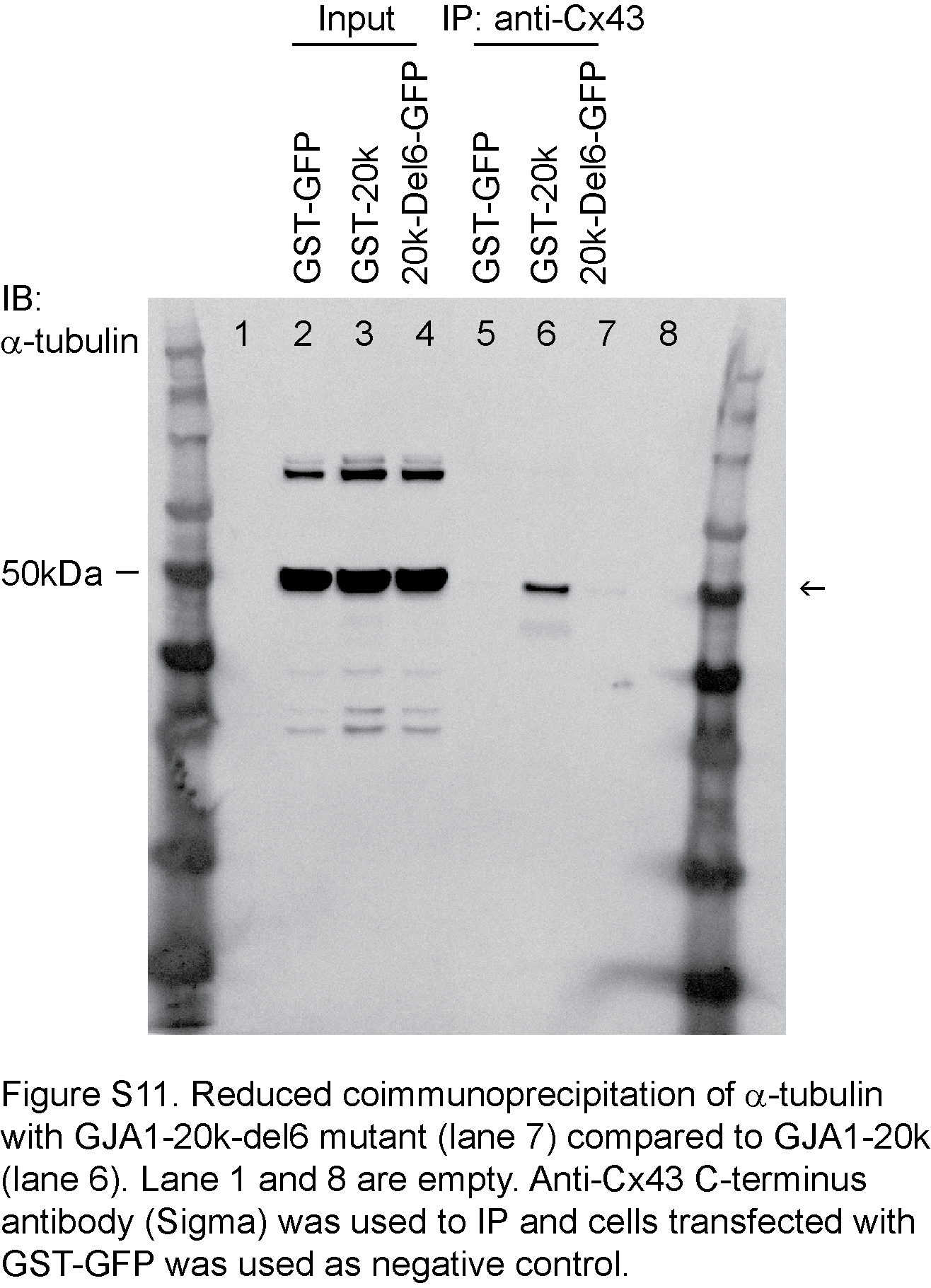

Supplement: Supplementary file 11 [file Image11.TIF]
