# Supplementary material for: Cx43 Isoform GJA1-20k Promotes Microtubule Dependent Mitochondrial Transport
Source: Front Physiol. 2017 Nov 7;8:905. doi: 10.3389/fphys.2017.00905 (PMC5682029; doi:10.3389/fphys.2017.00905)
Supplement: Supplementary file 17 [file Presentation1.PDF]

*Supplementary Material*

# Cx43 isoform GJA1-20k promotes microtubule dependent mitochondrial transport

Ying Fu[1*], Shan-Shan Zhang[1*], Shaohua Xiao[1*], Wassim A. Basheer[1], Rachel Baum[1], Irina Epifantseva[1], TingTing Hong[1,2], Robin M. Shaw[1,2,#]

**Correspondence:** Robin Shaw, MD. Ph.D: Robin.Shaw@cshs.org

## Supplemental Figure Legends

**Figure S1. GJA1-20k is localized to ER and extra-ER organelles.**
**(A)** Internal translation initiation in the *GJA1* mRNA produces the full-length GJA1-43k gap junction protein as well as the N-terminally truncated GJA1-20k isoform. **(B)** Confocal image of a HeLa cell transfected with GJA1-20k-GFP, immunolabeled for GFP (green) and the ER marker PDI (red). Arrows indicate clusters of GFP-positive signals. **(C)** Magnified area of the region outlined in B. Areas of colocalization (arrowheads) and association (arrows) between GFP and PDI are indicated. Pearson's coefficient = 0.722. Scale bar = 5 μm.

**Figure S2. Mitochondrial localization of endogenous small isoform GJA1-20k and full length Cx43.** C33A cells with endogenous Cx43 signal identified in green with an anti-Cx43 C-terminus antibody (top row) and anti Cx43 N-terminus antibody (bottom row). Only the anti C-terminus antibody localizes primarily to mitochondria, indicating isoforms smaller than full length Cx43 dominate mitochondrial signal. Scale bar = 5 mm.

**Figure S3. Mitochondria stretch along microtubules in cells with exogenous GJA1-20k.**
Transmission electron microscopy images of mitochondria (M) and microtubules (red arrows) in cells with exogenous GST-GFP versus GJA1-20k-GFP. Scale bar = 0.5 μm. *See also Figure 2C in the main text.*

**Figure S4. Microtubule-binding domain of GJA1-20k.**
GJA1-43k and GJA1-20k contain a putative microtubule-binding domain (MTBD, highlighted in blue). GJA1-20k protein sequence with internal start sites mutated to leucine (L). *See also Figure 3 in the main text.*

**Figure S5. Histograms of mitochondrial movements**
Original histograms of mitochondrial movements for each plasmid and condition that were used for the curve fits contained in Figure 3F.

**Figure S6. GJA1-20k maintains peripheral mitochondria, limiting fragmentation upon oxidative stress.**
Mitochondria morphology and distribution (Tom20 immunofluorescence, green) in HeLa cells expressing GFP-tagged GST, GJA1-43k, or GJA1-20k (red), treated with PBS or 300 μM $H_2O_2$

for 4 hours. Nuclei are marked by DAPI (blue). Cell borders are outlined by dashed blue lines. Regions used for Figure 4A are outlined in white. Scale bar = 5μm. *See also Figure 4A in the main text.*

**Figure S7. Full blot for Figure 1B upper panel (GJA1-43kHA).** Lane 4 is supernatant after first centrifuge at 1300g for 3 minutes. Lane 5 is an empty lane to space out before fractions obtained from percoll gradient.

**Figure S8, Full blot for Figure 1B lower panel (GJA1-20kHA).** Lane 4 is supernatant after first centrifuge at 1300g for 3 minutes. Lane 8 is suspension from cell pellet after initial centrifugation at 1300g.

**Figure S9. Full blot of Figure 2A.**

**Figure S10. Quantification of fluorescence ratio of peripheral/central mitochondria labeled by Tom20.** (A) Nucleus (N) ROI was defined by DAPI staining. The inner (green) ROI was drawn after thresholding at 1/6 of the maximal Tom20 intensity of the entire cell to define perinuclear region, and then further expanded by 4 micrometer to further include Golgi region (middle ROI, red). Signal from the nuclear ROI was removed from the cell as shown in (B). The peripheral ROI is defined from cell surface to the red ROI. The central ROI is defined from red ROI to nucleus ROI (purple) (B, C-shaded).

**Figure S11. Reduced coimmunoprecipitation of α-tubulin with GJA1-20k-del6 mutant (lane 7) compared to GJA1-20k (lane 6).** Lane 1 and 8 are empty. Anti-Cx43 C-terminus antibody (Sigma) was used for IP and cells transfected with GST-GFP was used as negative control.

**Supplemental Videos**

All videos are uncompressed AVI files compiled in Image J.

**Supplemental video 1.** Examples of GJA1-20k-mCherry (green) tracking along microtubules (red) marked by $\alpha$-tubulin-GFP. Original magnification: x100. Images were taken every 2.5 seconds for a total of 45 seconds. *See also Figure 2B in the main text.*

**Supplemental video 2.** Representative mitochondrion (mito-BFP) tracking in HeLa cells expressing GST, treated with DMSO. Original magnification: x100. Images were taken every 5 seconds for a total of 5 minutes. *See also Figure 3C-F in the main text.*

**Supplemental video 3.** Representative mitochondrion (mito-BFP) tracking in HeLa cells expressing GST, treated with nocodazole. Original magnification: x100. Images were taken every 5 seconds for a total of 5 minutes. *See also Figure 3C-F in the main text.*

**Supplemental video 4.** Representative mitochondrion (mito-BFP) tracking in HeLa cells expressing GJA1-20k, treated with DMSO. Original magnification: x100. Images were taken every 5 seconds for a total of 5 minutes. *See also Figure 3C-F in the main text.*

**Supplemental video 5.** Representative mitochondrion (mito-BFP) tracking in HeLa cells expressing GJA1-20k-del6, treated with DMSO. Original magnification: x100. Images were taken every 5 seconds for a total of 5 minutes. *See also Figure 3C-F in the main text.*
